# A meta analysis of the acupoint catgut embedding in the treatment of functional constipation

**DOI:** 10.3389/fmed.2025.1592220

**Published:** 2025-08-20

**Authors:** Yufei Zhao, Zhiwei Wang, Shangke Kuang, Shuxin Zhang

**Affiliations:** ^1^Department of Anorectal, Beijing University of Chinese Medicine, Beijing, China; ^2^Department of Anorectal, Dongzhimen Hospital, Beijing University of Chinese Medicine, Beijing, China

**Keywords:** acupoint catgut embedding, functional constipation, meta-analysis, randomised controlled trial, evidence-based medicine

## Abstract

**Objective:**

To evaluate the efficacy and safety of acupoint embedding for FC by meta-analysis, in order to provide evidence-based medical evidence for clinical practice.

**Methods:**

A comprehensive literature search was conducted in China National Knowledge Infrastructure, WanFang, VIP, PubMed, Web of Science and The Cochrane Library for randomised controlled trials (RCTs) on ACE for FC published from inception to November 2024. The included studies were assessed for quality using the modified Jadad scale, and statistical analysis was performed using RevMan 5.4.1 and Stata BE 17.

**Results:**

A total of 23 studies involving 1,794 patients were included. The meta-analysis showed that the total effective rate of ACE was significantly higher compared with oral Western medicine (odds ratio [OR] = 2.71, 95% confidence interval [CI]: 1.91–3.83, *p* < 0.00001), acupuncture (OR = 2.90, 95% CI: 1.68–5.01, *p* = 0.0001) and placebo groups (*p* < 0.05). There was no significant difference between ACE and oral Chinese medicine (OR = 2.34, 95% CI: 0.79–6.89, *p* = 0.12). The incidence of adverse reactions in the ACE group was low, presenting mainly as mild local discomfort such as soreness, bruising and pain, which were self-limiting.

**Conclusion:**

Acupoint catgut embedding demonstrates superior clinical efficacy compared with Western medicine, acupuncture and placebo in treating FC, with a low incidence of adverse effects. However, due to the limitations of the included studies, high-quality, large-sample RCTs are still needed to verify the long-term efficacy and safety of ACE.

**Systematic review registration:**

Identifier INPLASY202570017.

## 1 Introduction

Functional constipation (FC) is a functional gastrointestinal disorder without identifiable organic lesions. Patients with FC may generally present with defecation difficulties, a decreased frequency of bowel movements and a sensation of incomplete evacuation. These symptoms significantly impair the quality of life for affected individuals (1). In addition to elevating the risk of developing colorectal polyps, constipation may also serve as one of the underlying triggers for cardio-cerebrovascular events.

According to an existing epidemiological study, the global prevalence of constipation ranges from approximately 3 to 21% (2). The rapid pace of life, coupled with rising life pressures and alterations in dietary patterns, has led to a year-on-year increase in the number of patients seeking medical treatment for FC in China. As such, FC has emerged as a significant public health concern (3).

In recent years, acupuncture, as a component of traditional Chinese medicine (TCM), has demonstrated distinct advantages in the treatment of functional gastrointestinal disorders, attributable to its holistic regulatory effects. Acupoint catgut embedding (ACE), a significant branch of acupuncture, has gained widespread application in the treatment of FC. This method is favoured for its simple operation, lasting efficacy and high patient compliance. Acupoint catgut embedding has been documented in clinical studies to have certain therapeutic effects on FC symptoms. However, there is still insufficient evidence-based medical proof of its efficacy. Moreover, there is a notable absence of high-quality systematic reviews on this topic.

Accordingly, on the basis of a systematic review and meta-analysis of randomised controlled trials (RCTs) published up to November 2024, this study aims to evaluate the efficacy and safety of acupoint embedding for FC by meta-analysis, in order to provide evidence-based medical evidence for clinical practice.

## 2 Data and methods

### 2.1 Types of literature

#### 2.1.1 Types of research

This study was conducted based on the inclusion of RCTs and controlled clinical trials (CCTs) from both domestic and international sources. PRISMA guidelines were followed for this study. The registration number is INPLASY202570017.

#### 2.1.2 Inclusion criteria

To be included in the meta-analysis, studies had to meet the following criteria:

(1)  Publication date: RCTs or CCTs on ACE for FC published from inception to November 2024.
(2)  Participants: patients clearly diagnosed with FC [met Rome (II, III, IV) diagnostic criteria for FC (4)], irrespective of gender or age.
(3)  Interventions: The experimental group received ACE as a primary intervention, either alone or combined with other treatments.
(4)  Control measures: The control group was provided with oral Western medicine, standard acupuncture, oral TCM or a placebo.
(5)  Outcome measures: At a minimum, the total response rate must be reported (see Table 1). Additional efficacy evaluation indicators may include constipation severity scores using a constipation assessment scale (CAS), defecation frequency, stool consistency scores and quality of life scores using the Patient Assessment of Constipation Quality of Life (PAC-QOL) questionnaire.

**Table 1:** Criteria for efficacy evaluation of included literatures.

Author and publication date	Criteria for response evaluation
Tian Lijun 2021 (5)	According to the Consensus Opinions on the Diagnosis and Treatment of Functional Constipation with Integrated Traditional Chinese and Western Medicine, the efficacy index = (score before treatment − score after treatment)/score before treatment × 100%. (1) Ineffective: efficacy index <30%, the main symptoms and signs of patients were not improved or even aggravated; (2) Effective: 30% ≤ efficacy index <70%, the main symptoms and signs of patients were improved; (3) Significantly effective: 70% ≤ efficacy index <90%, the main symptoms and signs of patients were significantly improved; (4) Clinical bed healing: the number of therapeutic effect index ≥90%, the main symptoms and signs of patients basically disappeared or disappeared. Total clinical effective rate = (effective + markedly effective + clinical recovery)/total number of cases × 100%.
Liu Yang 2020 (6)	Significantly effective: all symptoms basically disappeared. Effective: the symptoms were significantly improved, and the frequency of symptoms was significantly reduced compared with that before treatment. Ineffective: no improvement of symptoms before and after treatment. Overall clinical response rate = (effective + markedly effective)/total number of cases × 100%.
Jiang Chunyan 2020 (7)	Refer to “TCM Syndrome Diagnosis Efficacy Criteria.” Recovered: normal stool, all other symptoms disappeared. Significantly effective: constipation significantly improved, with interval time and stool quality close to normal; or slightly dry stool with defecation interval time within 72 h, most other symptoms disappeared, and the score decreased 2/3. Effective: decreased defecation interval 1 days, or dry stool improved, other symptoms were improved, and scores decreased l/3, but not 2/3. Ineffective: no improvement in constipation and other symptoms or insufficient reduction in integral value 1/3. Overall clinical response rate = (effective + significant effect + clinical healing)/total number of cases × 100%.
Li Shiying 2020 (8)	Refer to “TCM Syndrome Diagnosis Efficacy Criteria.” Recovered: normal stool, all other symptoms disappeared. Significantly effective: constipation significantly improved, with interval time and stool quality close to normal; or slightly dry stool with defecation interval time within 72 h, most other symptoms disappeared, and the score decreased 2/3. Effective: decreased defecation interval 1 days, or dry stool improved, other symptoms were improved, and scores decreased l/3, but not 2/3. Ineffective: no improvement in constipation and other symptoms or insufficient reduction in integral value 1/3. Overall clinical response rate = (effective + significant effect + clinical healing)/total number of cases × 100%.
Zhang Xiaohui 2019 (9)	Refer to “TCM Syndrome Diagnosis Efficacy Criteria.” Recovered: normal stool, all other symptoms disappeared. Significantly effective: constipation significantly improved, with interval time and stool quality close to normal; or slightly dry stool with defecation interval time within 72 h, most other symptoms disappeared, and the score decreased 2/3. Effective: decreased defecation interval 1 days, or dry stool improved, other symptoms were improved, and scores decreased l/3, but not 2/3. Ineffective: no improvement in constipation and other symptoms or insufficient reduction in integral value 1/3. Overall clinical response rate = (effective + significant effect + clinical healing)/total number of cases × 100%.
Liu Anli 2019 (10)	Significantly effective: After treatment, the patient can defecate, defecate at least once within 2 days, and the clinical symptoms completely disappear. Effective: After treatment, the patient can defecate, defecate at least once within 3 days, and the clinical symptoms are basically improved. Ineffective: After treatment, the patient’s constipation symptoms were not improved, and the condition was aggravated. Overall clinical response rate = (effective + significant)/total number of cases × 100%.
Du Binglin 2018 (11)	Refer to “TCM Syndrome Diagnosis Efficacy Criteria.” Recovered: normal stool, all other symptoms disappeared. Significantly effective: constipation significantly improved, with interval time and stool quality close to normal; or slightly dry stool with defecation interval time within 72 h, most other symptoms disappeared, and the score decreased 2/3. Effective: decreased defecation interval 1 days, or dry stool improved, other symptoms were improved, and scores decreased l/3, but not 2/3. Ineffective: no improvement in constipation and other symptoms or insufficient reduction in integral value 1/3. Overall clinical response rate = (effective + significant effect + clinical healing)/total number of cases × 100%.
Zhang Wenjing 2018 (12)	Refer to the relevant standards of “Diagnostic and Therapeutic Criteria for TCM Syndrome.” Recovered: defecation once within 2 days, stool quality is fair, defecation is smooth, no sense of incomplete evacuation; effective: defecation once within 3 days, stool quality turns moist, defecation is not smooth; improved: symptoms are improved than before, but not obvious; ineffective: still need to use stimulant laxatives or defecation frequency and stool quality are not improved. Overall clinical response rate = (effective + significant effect + clinical healing)/total number of cases × 100%.
Zhai Dong 2018 (13)	It is formulated by referring to Guidelines for Clinical Research of New Drugs of Traditional Chinese Medicine (Interim). Cure: normal stool, or return to premorbid levels, defecation > 3 times per week, other symptoms all disappeared. Improvement: constipation was significantly improved, the interval time and stool quality were close to normal or the stool was slightly dry while the defecation interval time was <72 h, and a large part of other symptoms disappeared. Ineffective: constipation and other symptoms were not improved. Overall clinical response rate = (improved + cured)/total number of cases × 100%.
Wang Peiyan 2016 (14)	Recovered: stool texture became soft, smooth when relieving, combined symptoms completely eliminated loss, defecation frequency within 5 days was more than 2 days, and lasted for more than 3 months. Significantly effective: slightly moist stool texture, defecation became unobstructed, combined symptoms were significantly improved, defecation frequency within 5 days was more than 2 days, lasting for less than 3 months. Effective: defecation is not smooth, stool texture is dry first and then soft, combined symptoms are not improved, defecation frequency within 5 days is more than 3 days once, lasting for less than 3 months, more than 15 days. Invalid: Criteria for “Valid” not met. Overall clinical response rate = (effective + significant effect + clinical healing)/total number of cases × 100%.
Zheng Wei 2016 (15)	According to nimodipine method to determine the efficacy of symptoms, constipation score scale was used to calculate the difference of scores before and after treatment for comparison between groups. Reduction rate = (score before treatment − score after treatment)/score before treatment × 100%. Recovered: score reduction rate 76–100%; markedly effective: score reduction rate 51–75%; effective: score reduction rate 25–50%; ineffective: score reduction rate <25%. Overall clinical response rate = (effective + significant effect + clinical healing)/total number of cases × 100%.
Liu Renghai 2015 (16)	Criteria for efficacy determination of TCM syndrome scores were formulated by referring to Guidelines for Clinical Research of New Drugs of Traditional Chinese Medicine. Efficacy index = [(score before treatment − score after treatment)/score before treatment] × 100%. Clinical recovery: normal stool, complete disappearance of main symptoms, or efficacy index of 100%. Significantly effective: normal stool or return to the pre-disease level, constipation significantly improved, interval time and stool quality close to normal, slightly dry stool and defecation time within 72 h, most other symptoms disappeared, or 100% > efficacy index ≥ 70%. Effective: shorten defecation time by 1 day, or dry stool improved, other symptoms were improved, or 70% > efficacy index > 30%. Ineffective: no improvement in constipation or other symptoms, or efficacy index ≤ 30%. Overall clinical response rate = (effective + significant effect + clinical healing)/total number of cases × 100%.
Wang Xiaolong 2014 (17)	Recovered: Defecation within 4 d of treatment, and defecation at least once every 2 days thereafter, symptoms disappeared, and the effect lasted for at least 3 months. Significantly effective: defecation within 4 days of treatment, and defecation at least once every 2 days thereafter, with efficacy lasting for more than 30 days, but less than 3 months. Effective: defecation within 4 days of treatment, and defecation at least once every 3 days thereafter, the effect lasts for 15 to 30 days. Ineffective: no significant improvement in symptoms. Overall clinical response rate = (effective + significant effect + clinical healing)/total number of cases × 100%.
Sun Yuanzheng 2014 (18)	Refer to the curative effect standard of constipation in the Diagnostic and Therapeutic Criteria for TCM Syndrome. Cure: defecation once within 2 d, stool quality turned moist, smooth during solution, accompanied by symptoms disappeared. Significantly effective: defecation within 2 days, stool quality turned moist, defecation was not smooth, accompanied by symptom relief. Effective: defecation within 3 d, dry and soft stool, poor defecation, accompanied by no relief of symptoms. Ineffective: constipation and accompanying symptoms did not improve. Overall clinical response rate = (effective + significant effect + clinical healing)/total number of cases × 100%.
Liao Rifeng 2014 (19)	Refer to “TCM Syndrome Diagnosis Efficacy Criteria.” Recovered: normal stool, all other symptoms disappeared. Significantly effective: constipation significantly improved, with interval time and stool quality close to normal; or slightly dry stool with defecation interval time within 72 h, most other symptoms disappeared, and the score decreased 2/3. Effective: decreased defecation interval 1 days, or dry stool improved, other symptoms were improved, and scores decreased l/3, but not 2/3. Ineffective: no improvement in constipation and other symptoms or insufficient reduction in integral value 1/3. Overall clinical response rate = (effective + significant effect + clinical healing)/total number of cases × 100%.
Qu Muwen 2013 (20)	It is planned to refer to the Interim Criteria for Diagnosis and Treatment of Constipation “formulated by the Anorectal Group of Chinese Society of Surgery. Cure: defecation frequency returned to normal, once every 1 ~ 2 days, stool character was normal, manifested as soft stool, defecation patency without difficulty, no residual sensation after defecation. Improvement: normal frequency of defecation, but unstable, feeling of incomplete evacuation after residual defecation. Ineffective: no significant improvement in various indicators. Overall clinical response rate = (cured + improved)/total number of cases × 100%.
Peng Hui 2013 (21)	Recovered: Defecation within 4 d of treatment, and defecation at least once every 2 days thereafter, symptoms disappeared, and the effect lasted for at least 3 months. Significantly effective: defecation within 4 days of treatment, and defecation at least once every 2 days thereafter, with efficacy lasting for more than 30 days, but less than 3 months. Effective: defecation within 4 days of treatment, and defecation at least once every 3 days thereafter, the effect lasts for 15 to 30 days. Ineffective: no significant improvement in symptoms. Overall clinical response rate = (effective + significant effect + clinical healing)/total number of cases × 100%.
Wu Shengzhi 2013 (22)	Cure: 1–2 d defecation once, soft and moist stool, defecation time ≤10 min, straining during defecation or defecation without feeling; markedly effective: 2–3 d defecation once, dry and soft stool, defecation time 10–20 min, occasional straining or defecation without feeling; effective: 3–4 d defecation once, dry and hard stool, defecation time 20–30 min, sometimes straining or defecation without feeling; ineffective more than 5d defecation once, hard stool, defecation > 30 min, straining during defecation. Overall clinical response rate = (effective + significant effect + clinical healing)/total number of cases × 100%.
Ding Jie 2011 (23)	Recovered: Defecation within 4 d of treatment, and defecation at least once every 2 days thereafter, symptoms disappeared, and the effect lasted for at least 3 months. Significantly effective: defecation within 4 days of treatment, and defecation at least once every 2 days thereafter, with efficacy lasting for more than 30 days, but less than 3 months. Effective: defecation within 4 days of treatment, and defecation at least once every 3 days thereafter, the effect lasts for 15 to 30 days. Ineffective: no significant improvement in symptoms. Overall clinical response rate = (effective + significant effect + clinical healing)/total number of cases × 100%.
Fang Qingxia 2011 (24)	Refer to “TCM Syndrome Diagnosis Efficacy Criteria.” Recovered: normal stool, all other symptoms disappeared. Significantly effective: constipation significantly improved, with interval time and stool quality close to normal; or slightly dry stool with defecation interval time within 72 h, most other symptoms disappeared, and the score decreased 2/3. Effective: decreased defecation interval 1 days, or dry stool improved, other symptoms were improved, and scores decreased l/3, but not 2/3. Ineffective: no improvement in constipation and other symptoms or insufficient reduction in integral value 1/3. Overall clinical response rate = (effective + significant effect + clinical healing)/total number of cases × 100%.
Li Suhe 2011 (25)	Refer to “TCM Syndrome Diagnosis Efficacy Criteria.” Recovered: normal stool, all other symptoms disappeared. Significantly effective: constipation significantly improved, with interval time and stool quality close to normal; or slightly dry stool with defecation interval time within 72 h, most other symptoms disappeared, and the score decreased 2/3. Effective: decreased defecation interval 1 days, or dry stool improved, other symptoms were improved, and scores decreased l/3, but not 2/3. Ineffective: no improvement in constipation and other symptoms or insufficient reduction in integral value 1/3. Overall clinical response rate = (effective + significant effect + clinical healing)/total number of cases × 100%.
Wang Wenjun 2011 (26)	Refer to “TCM Syndrome Diagnosis Efficacy Criteria.” Recovered: normal stool, all other symptoms disappeared. Significantly effective: constipation significantly improved, with interval time and stool quality close to normal; or slightly dry stool with defecation interval time within 72 h, most other symptoms disappeared, and the score decreased 2/3. Effective: decreased defecation interval 1 days, or dry stool improved, other symptoms were improved, and scores decreased l/3, but not 2/3. Ineffective: no improvement in constipation and other symptoms or insufficient reduction in integral value 1/3. Overall clinical response rate = (effective + significant effect + clinical healing)/total number of cases × 100%.
Xiao Guang 2010 (27)	Refer to “TCM Syndrome Diagnosis Efficacy Criteria.” Recovered: normal stool, all other symptoms disappeared. Significantly effective: constipation significantly improved, with interval time and stool quality close to normal; or slightly dry stool with defecation interval time within 72 h, most other symptoms disappeared, and the score decreased 2/3. Effective: decreased defecation interval 1 days, or dry stool improved, other symptoms were improved, and scores decreased l/3, but not 2/3. Ineffective: no improvement in constipation and other symptoms or insufficient reduction in integral value 1/3. Overall clinical response rate = (effective + significant effect + clinical healing)/total number of cases × 100%.

#### 2.1.3 Exclusion criteria

This study excluded literature that met any of the following criteria:

(1)  Constipation caused by organic lesions (e.g., intestinal obstruction, inflammatory bowel disease and tumours).
(2)  Patients with concurrent psychiatric disorders (e.g., severe anxiety and depression).
(3)  Pregnant or lactating women.
(4)  Acupoint catgut embedding was used only as a partial or temporary treatment, or was also used in the control group.
(5)  Studies with incomplete data or studies from which valid data could not be extracted.

### 2.2 Literature retrieval strategy

A computer-based retrieval of eligible studies was conducted in the China National Knowledge Infrastructure (CNKI), WanFang Data, VIP, PubMed, Web of Science and Cochrane Library databases. The time for the retrieval was from the inception of databases until November 2024 (see Attachment 1).

Search terms:

- Chinese: Constipation, functional constipation, slow transit constipation, defecation disorder constipation, functional defecation disorder, catgut embedding at acupoints, randomised, randomised controlled.
- English: Constipation, functional constipation, slow transit constipation, defecation disorder constipation, functional defecation disorder, catgut embedding at acupoints, randomised, randomised controlled.

The retrieval strategy integrated free-text terms and Medical Subject Headings for refinement. Additionally, manual searches of reference lists from relevant articles were performed to identify any potentially overlooked studies.

Search methods: The first group of search terms included “catgut embedding” and “acupoint catgut embedding”, and each keyword was connected with “or” relationship. The second group of search terms included “constipation”, “functional constipation” and “slow transit constipation”, and each keyword was connected with “or” relationship. The third group of search terms included “random” and “randomised controlled”, and each keyword was connected with “or” relationship. The three groups of search terms were connected with “and” relationship, and the English search terms were searched with the same relationship at the same time.

### 2.3 Literature quality assessment

The methodological quality of each literature study included in the current research was evaluated using the modified Jadad scale:

- Studies with a score of 1–3 points were categorised as having poor quality.
- Studies with a score of 4–7 points were classified as having high quality.

The assessment covered aspects such as randomisation, allocation concealment, blinding, follow-up and dropout rates, ensuring the scientific rigour and reliability of the included literature.

### 2.4 Statistical methods

Data analysis was conducted using Review Manager (RevMan 5.4.1) and Stata BE 17 software for meta-analysis and plotting.

(1)  Heterogeneity test: The Q test and *I^2^* statistic were used to assess the heterogeneity between studies:

- The fixed-effect model was used when there was acceptable homogeneity (*p* ≥ 0.1 and *I^2^* < 50%).
- Otherwise, the random-effect model was selected in case of heterogeneity (*p* < 0.1 and *I^2^* ≥ 50%). Meanwhile, sensitivity analysis was conducted by sequentially omitting each study to identify the source of heterogeneity.

(2)  Categorical data: The odds ratio (OR) was used to assess efficacy alongside the calculation of 95% confidence intervals (95% CI). A *p* ≤ 0.05 was considered statistically significant.
(3)  Publication bias analysis: The possibility of publication bias was assessed using a funnel plot and Egger’s linear regression analysis. Any identified bias was adjusted using the trim and fill method.

## 3 Results

### 3.1 Literature retrieval results

**Figure 1:**
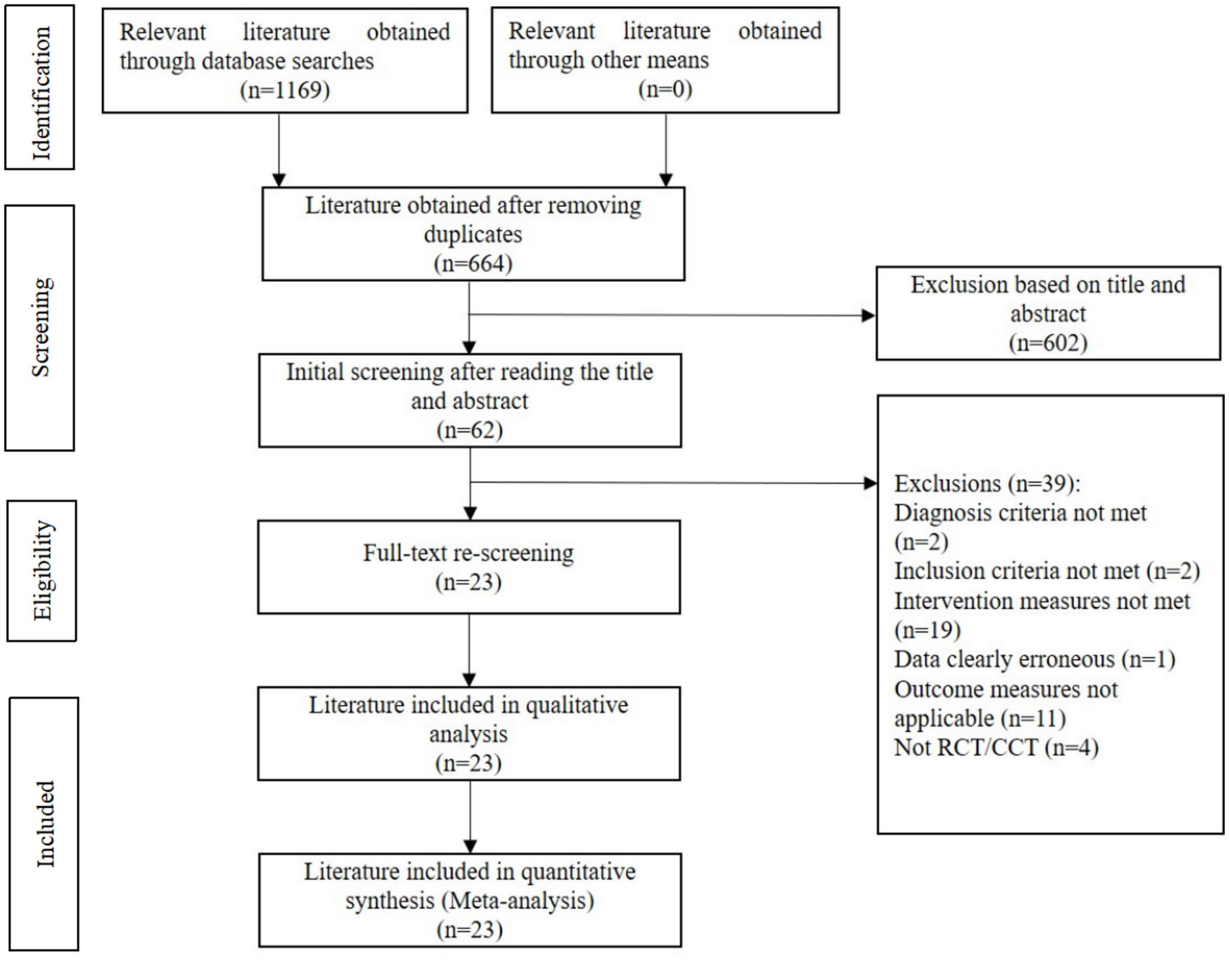
Literature screening flow chart.

A total of 1,169 relevant articles were retrieved in this study. Then, 505 duplicates were removed through literature management using EndNote. Based on the established inclusion and exclusion criteria, the titles and abstracts of the remaining articles were checked to exclude another 602 articles. Subsequent full-text reviews resulted in the final inclusion of 23 articles (published between 2010 and 2021) (5–27), involving 1,794 patients. The literature screening process is shown in Figure 1.

### 3.2 Basic characteristics of the included studies

A total of 23 articles were included, all of which were published in Chinese between 2010 and 2021. These articles involved 24 distinct groups (including the comparison of ACE with acupuncture and Western medicine separately by Zheng Wei et al. (15)), totalling 1,794 participants. Five studies were related to the comparison between an ACE group and a TCM group, involving 289 cases. Eleven studies conducted comparative analyses between an ACE group and a Western medicine group, with 954 cases. Meanwhile, another 7 studies (515 cases in total) compared an ACE group and an acupuncture group. Additionally, a comparison between an ACE group and a sham embedding (placebo) group was mentioned in 1 study with 60 cases.

### 3.3 Methodological quality rating of the included studies

Scoring results based on a modified Jadad scale are shown in Table 2. Among the 23 articles, 1 article scored 5 points, 1 scored 4 points, 12 scored 3 points, and 9 articles scored 2 points.

**Table 2:** Scoring results based on modified Jadad scale.

Author and publication date	Quality assessment and Jadad score	Subjects	Intervention measures	Treatment outcomes		Dropouts and loss to follow-up
				Sample size total effective [*n* (%)]		
Tian Lijun 2021 (5)	The study used a random number table method without implementation of allocation concealment or blinding.	Experimental group	ACE	58	55 (94.83)	Not mentioned
	Jadad score: 3 points	Control group	Mosapride citrate tablets	60	45 (75.00)	
Liu Yang 2020 (6)	The study used a random number table method without implementation of allocation concealment or blinding.	Experimental group	ACE	30	28 (93.3)	Not mentioned
	Jadad score: 3 points	Control group	Acupuncture	30	22 (73.3)	
Jiang Chunyan 2020 (7)	Participants were randomly divided into two groups without allocation concealment or blinding.	Experimental group	ACE	21	19 (90.5)	Not mentioned
	Jadad score: 2 points	Control group	Acupuncture	21	17 (81.0)	
Li Shiying 2020 (8)	This study utilized a random number table method with allocation concealment but without blinding.	Experimental group	ACE	30	23 (76.67)	There were no dropouts or withdrawals mentioned.
	Jadad score: 5 points	Control group	Mosapride citrate tablets	30	17 (56.67)	
Zhang Xiaohui 2019 (9)	The study used a random number table method without implementation of allocation concealment or blinding.	Experimental group	ACE	45	39 (86.7)	Not mentioned
	Jadad score: 3 points	Control group	Acupuncture	45	29 (64.4)	
Liu Anli 2019 (10)	The study used a random number table method without implementation of allocation concealment or blinding.	Experimental group	ACE	49	47 (95.9)	Not mentioned
	Jadad score: 3 points	Control group	Mosapride citrate tablets	46	35 (76.1)	
Du Binglin 2018 (11)	Participants were randomly divided into two groups without allocation concealment or blinding.	Experimental group	ACE	30	26 (86.7)	Not mentioned
	Jadad score: 2 points	Control group	Polyethylene glycol 4,000 powder	30	18 (60.0)	
Zhang Wenjing 2018 (12)	Participants were randomly divided into two groups without allocation concealment or blinding.	Experimental group	ACE	30	28 (93.33)	Not mentioned
	Jadad score: 2 points	Control group	Placebo (sham embedding)	30	13 (43.44)	
Zhai Dong 2018 (13)	Participants were randomly divided into two groups without allocation concealment or blinding.	Experimental group	ACE	25	21 (84.0)	5 cases in the experimental group
	Jadad score: 2 points	Control group	Lactulose oral solution	27	19 (70.37)	3 cases in the control group,
						Reasons for dropout not described
Wang Peiyan 2016 (14)	Participants were randomly divided into two groups without allocation concealment or blinding.	Experimental group	ACE	11	10 (90.9)	Not mentioned
	Jadad score: 2 points	Control group	Acupuncture	10	8 (80.0)	
Zheng Wei 2016 (15)	Participants were randomly divided into two groups without allocation concealment or blinding.	Experimental group	ACE	24	20 (83.3)	Not mentioned
	Jadad score: 2 points	Control group 1	Acupuncture	24	19 (79.2)	
		Control group 2	Polyethylene glycol 4,000 powder	24	18 (75.0)	
Liu Renghai 2015 (16)	Participants were randomly divided into two groups without allocation concealment or blinding.	Experimental group	ACE	90	72 (80.0)	Not mentioned
	Jadad score: 2 points	Control group	Mosapride citrate tablets	90	62 (68.9)	
Wang Xiaolong 2014 (17)	The study used a random number table method without implementation of allocation concealment or blinding.	Experimental group	ACE	30	28 (93.33)	Not mentioned
	Jadad score: 3 points	Control group	Cannabis fruit spleen-enriching pill	31	23 (74.20)	
Sun Yuanzheng 2014 (18)	The study used a random number table method without implementation of allocation concealment or blinding.	Experimental group	ACE	32	31 (96.9)	Not mentioned
	Jadad score: 3 points	Control group	Phenolphthalein tablets	32	27 (84.4)	
Liao Rifeng 2014 (19)	The study used a random number table method without implementation of allocation concealment or blinding.	Experimental group	ACE	30	22 (73.33)	Not mentioned
	Jadad score: 3 points	Control group	Modified astragalus decoction	30	24 (80.00)	
Qu Muwen 2013 (20)	The study used a random number table method without implementation of allocation concealment or blinding.	Experimental group	ACE	30	27 (90.0)	Not mentioned
	Jadad score: 3 points	Control group	Mosapride citrate tablets	30	26 (86.7)	
Peng Hui 2013 (21)	The study used a random number table method without implementation of allocation concealment or blinding.	Experimental group	ACE	32	30 (93.75)	Not mentioned
	Jadad score: 3 points	Control group	Cannabis fruit spleen-enriching pill	30	21 (70.00)	
Wu Shengzhi 2013 (22)	Participants were randomly divided into two groups without allocation concealment or blinding.	Experimental group	ACE	49	47 (95.92)	There were no dropouts or withdrawals mentioned.
	Jadad score: 3 points	Control group	Polyethylene glycol 4,000 powder	48	37 (77.08)	
Ding Jie 2011 (23)	The study used a stratified random sampling method, without implementing randomisation concealment and blinding.	Experimental group	ACE	50	48 (96.0)	Not mentioned
	Jadad score: 2 points	Control group	Acupuncture	60	57 (95.0)	
Fang Qingxia, 2011 (24)	The study used a random number table method without implementation of allocation concealment or blinding.	Experimental group	ACE	72	68 (94.44)	Not mentioned
	Jadad score: 3 points	Control group	Acupuncture	72	58 (80.56)	
Li Suhe 2011 (25)	The study used a random number table method without implementation of allocation concealment or blinding.	Experimental group	ACE	32	26 (81.2)	Not mentioned
	Jadad score: 3 points	Control group	Maren soft capsules	31	26 (83.9)	
Wang Wenjun 2011 (26)	Participants were randomly divided into two groups without allocation concealment or blinding.	Experimental group	ACE	60	50 (83.33)	Not mentioned
	Jadad score: 2 points	Control group	Polyethylene glycol 4,000 powder	60	45 (75.00)	
Xiao Guang 2010 (27)	This study utilized a random number table method with allocation concealment but without blinding.	Experimental group	ACE	21	20 (95.24)	Not mentioned
	Jadad score: 4 points	Control group	Tongbianling capsules	22	15 (68.18)	

### 3.4 Meta-analysis of clinical efficacy

#### 3.4.1 Comparison between the acupoint catgut embedding group and the traditional Chinese medicine group

**Figure 2:**
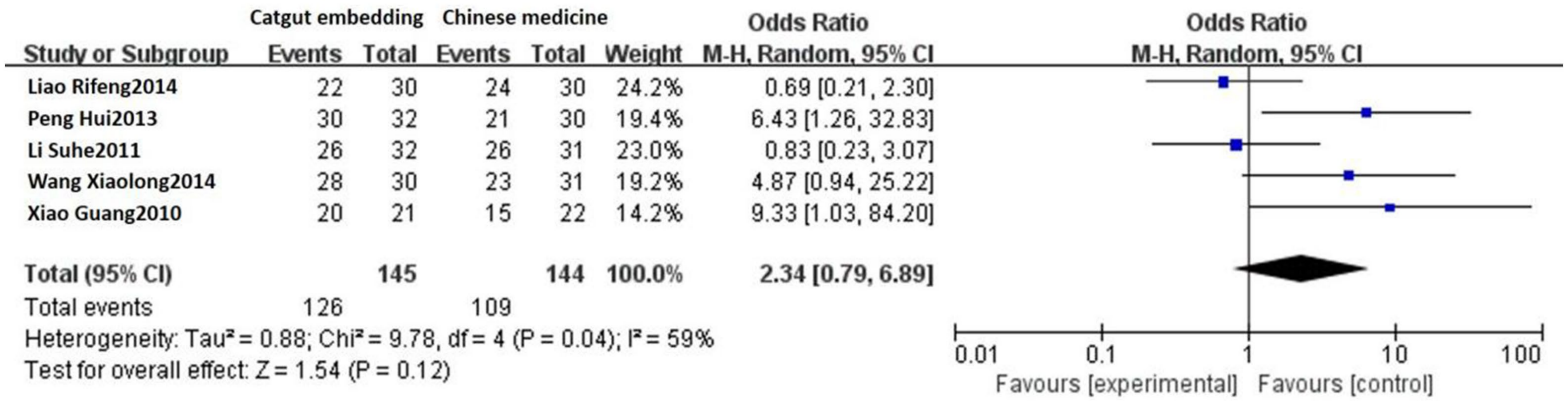
Forest plot comparing ACE and TCM.

Five studies reported the response rates of ACE vs. TCM. Heterogeneity was detected among these studies (*p* = 0.04, *I^2^* = 59%). Sensitivity analysis indicated that the high heterogeneity was due to the negative results reported in the study by Liao Rifeng et al. (19), which was not excluded after a thorough reading. Using a random-effects model, the results showed that the response rate was 86.90% (126/145) in the ACE group and 75.69% (109/144) in the TCM group. As shown in Figure 2, there was no statistically significant difference between the two groups [OR = 2.34, 95% CI: 0.79, 6.89, Z = 1.54, *p* = 0.12].

#### 3.4.2 Comparison between the acupoint catgut embedding group and Western medicine group

**Figure 3:**
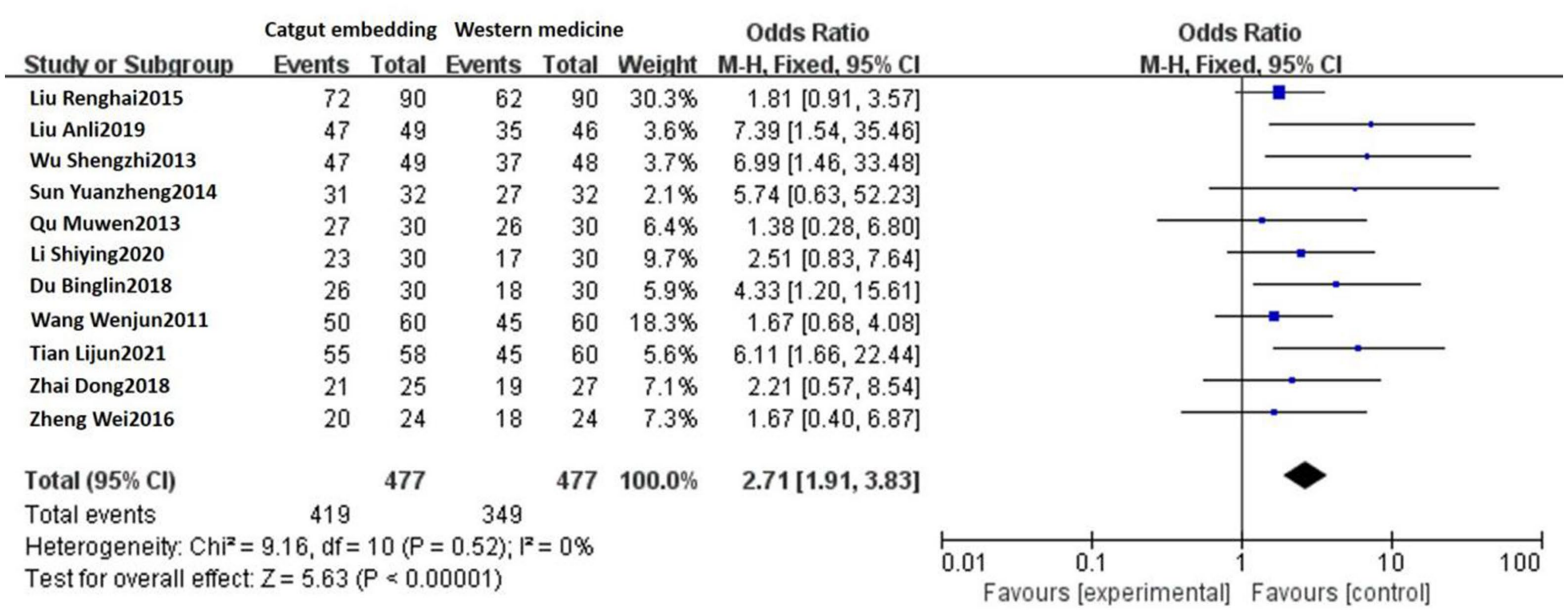
Forest plot comparing ACE and Western medicine.

Eleven studies reported the response rates of ACE vs. Western medicine. No heterogeneity was detected among these studies (*p* = 0.52, *I^2^* = 0%), and a fixed-effects model was used. The results showed that the response rates were 87.84% (419/477) and 73.17% (349/477) in the ACE group and the Western medicine group, respectively. There was a statistically significant difference between the two groups [OR = 2.71, 95% CI: 1.91, 3.83, Z = 5.63, *p* < 0.00001; see Figure 3].

#### 3.4.3 Comparison between the acupoint catgut embedding group and the acupuncture group

**Figure 4:**
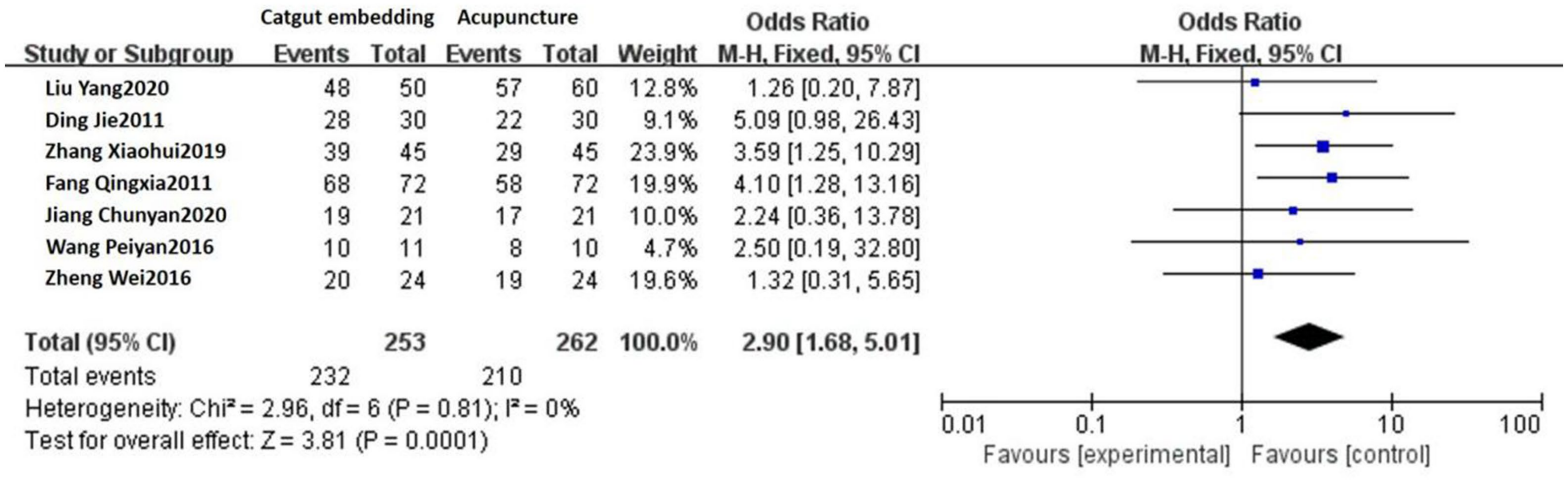
Forest plot comparing ACE and acupuncture.

Seven studies reported the response rates of ACE vs. acupuncture. A fixed-effect model was used, considering that no heterogeneity was detected among these studies (*p* = 0.81, *I^2^* = 0%). The response rate of the ACE group was 91.70% (232/253) and 80.15% (210/262) in the acupuncture group. There was a statistically significant difference between the two groups [OR = 2.90, 95% CI: 1.68, 5.01, Z = 3.81, *p* = 0.0001; see Figure 4].

#### 3.4.4 Comparison between the acupoint catgut embedding group and a sham embedding (placebo) group

One study reported the response rates of ACE and sham embedding. The response rate of ACE was 93.33% (28/30), while that of sham embedding was 43.33% (13/30). The response rate of ACE was higher compared with sham embedding, and the difference was significant (Z = 3.55, *p* = 0.0004).

### 3.5 Bias analysis

**Figure 5:**
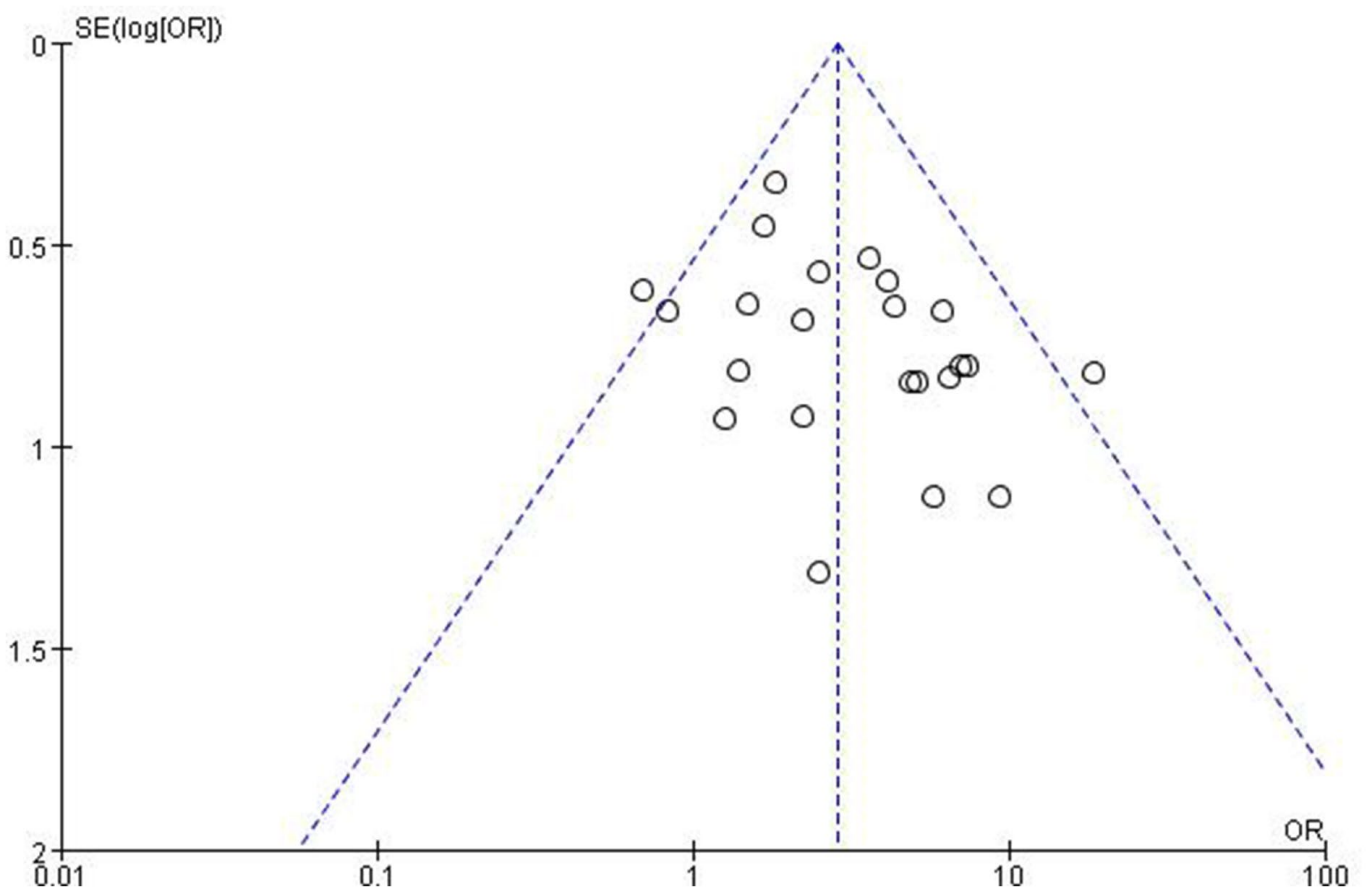
Publication bias, funnel plot.

**Figure 6:**
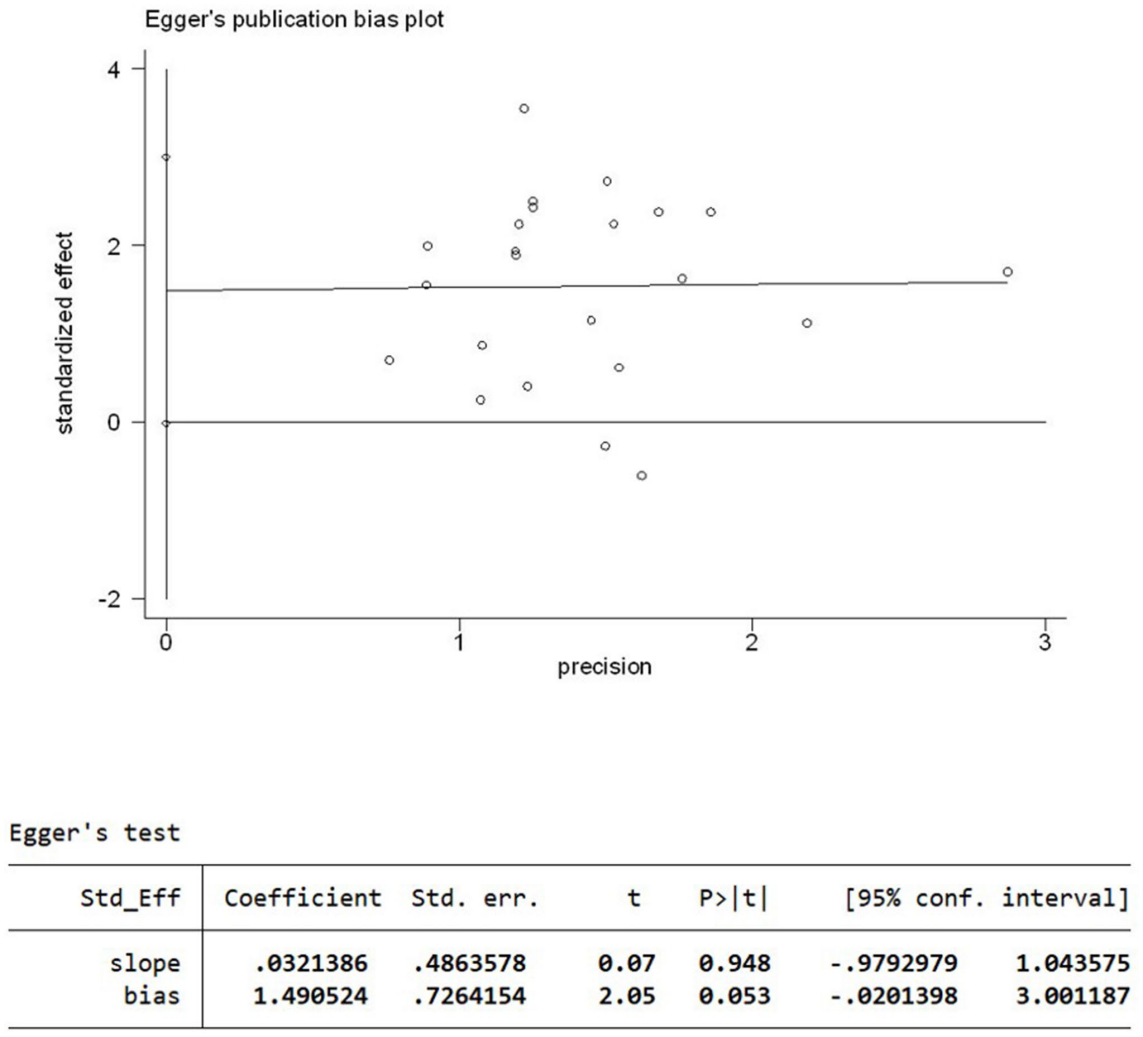
Egger’s linear regression plot.

A funnel plot (Figure 5) of response rates was drawn with the OR on the X-axis and standard error on the Y-axis, and the scatter plot was essentially symmetrical. No significant publication bias was observed according to the results of Egger’s linear regression as shown in Figure 6 (*p* = 0.053 > 0.05).

### 3.6 Adverse reactions

Among the 23 articles reviewed, 7 articles specifically documented adverse reactions, which were observed in 5 cases among 296 patients in the experimental group. The reported adverse reactions included localised soreness, swelling, bruising and pain at the site of the catgut embedding. All of these symptoms were effectively alleviated following the application of a local hot compress.

## 4 Discussion

Functional constipation falls into the category of “constipation” in TCM and is characterised by an ease of diagnosis but difficulty in treatment, with its pathophysiological mechanisms not yet fully elucidated. In modern medicine, FC is commonly treated by pharmacological agents, including laxatives, prokinetic agents and microbiological preparations, as well as interventions such as enemas and surgical procedures. Nevertheless, these methods exhibit certain limitations concerning their long-term efficacy and safety (28). The prolonged use of stimulant laxatives, including anthraquinone drugs, aloe, senna leaves and rhubarb, may be associated with an elevated risk of melanosis coli and colon polyps (29). Against this background, a strong focus has been attached to the benefits of ACE therapy, grounded in TCM theory, in the treatment of constipation. At present, ACE therapy for constipation presents a mature clinical system in Asia, especially in China, and is also widely used in South Korea and Japan. However, its popularity is lower in China, and it is a niche therapy in Europe and the United States, where it is still in the research and exploration stage.

According to *Miraculous Pivot: End and Beginning*, “For chronic diseases, where pathogenic factors have penetrated deeply, needles should be inserted deeply and retained for longer periods”. This emphasises that for chronic conditions, acupuncture must reach deep into the affected area and provide sustained action. Acupoint catgut embedding has been developed in accordance with this theoretical framework (30). The treatment is the product of combining acupuncture and moxibustion theory, Chinese *materia medica* and modern physics. Under the guidance of acupuncture and the moxibustion meridian system, catgut or other absorbable sutures are placed in the corresponding acupoint area. Following a soft, slow, long-term and benign acupoint acupuncture stimulation effect, it achieves the effect of dredging the meridians, qi and blood, and is able to prevent and treat diseases (30). It is a compound treatment method that combines multiple therapies and multiple effects. From the perspective of TCM, the acupoints for catgut embedding (e.g., Tianshu, Dachangshu, and Zusanli) can invigorate the spleen and qi, promote the transport of the spleen and stomach and restore intestinal conduction function. Catgut embedding can also stimulate dredging of the meridians and promote the patency of qi and blood (31). From a Western medicine perspective, the embedded suture-body serves as a form of minor foreign body stimulation at acupoints, continuously excites local nerves (e.g., the intestinal plexus), promotes intestinal smooth muscle contraction and accelerates defecation (32). In addition, ACE has been found to improve serum brain–gut peptide levels in patients with slow transit constipation (33), reduce neuronal apoptosis and, subsequently, relieve constipation symptoms (34). Compared to traditional acupuncture, catgut utilised in ACE is typically fully absorbed within a period of 14 to 21 days. Throughout this duration, it provides continuous stimulation to the acupoints, thereby reducing the frequency of medical visits to some extent and enhancing patient compliance with treatment (35). Acupoint catgut embedding has gained widespread application in clinical practice due to its prolonged stimulation, user-friendly operation and lasting therapeutic effects. Based on the included literature, this study concludes that the commonly used acupoints of ACE for the treatment of constipation include Guanyuan, Zusanli and Dachangshu, which regulate intestinal function, increase the frequency of defecation and improve the condition of faeces by stimulating these acupoints. Xue Qiming et al. (36) performed ACE at the Tianshu and Abdominal Jie points, and the results showed that the weekly average complete defecation frequency increased in the catgut embedding group after treatment, and the PAC-QOL quality of life score was lower after catgut embedding treatment than in the control group, indicating that ACE could achieve the effect of regulating large intestine fu-qi. In a study conducted by Yin Ping (37), catgut embedding was performed at the Shangjuxu, Tianshu, Zusanli, Shuidao and Dachangshu points, and the overall response rate was 83.3% after treatment. The symptom checklist PAC-SYM, PAC-QOL and straining during defecation and abdominal distension scores were significantly improved.

In the present meta-analysis, ACE showed significant clinical efficacy in the treatment of FC. The overall response rate was markedly superior to that of oral Western medicine, standard acupuncture and placebo (sham embedding) treatments, and its efficacy was comparable to that of oral TCM. Furthermore, there was a relatively low incidence of adverse reactions associated with ACE, primarily presenting as localised soreness, swelling, bruising and mild pain when present. These symptoms can typically be mitigated through the application of a local hot compress. Notably, there were no documented cases of severe adverse events, suggesting a favourable safety profile for this therapy. There are, however, some caveats to remember when performing ACE therapy. First, to avoid infection, following catgut embedding at acupoints, the embedding site should not be contaminated with water, but complexed iodine can be used to disinfect the local area and keep it clean. Hot weather easy to sweat, at this time the catgut embedding time should not be too long to prevent infection. Second, the patient should be mindful of staying warm because once catgut embedding at acupoints has taken place, the embedding site will have subtle pinholes; cold stimulation should be avoided to not cause a decreased body resistance, which may affect the effect of catgut embedding at acupoints. Finally, diet should be monitored; spicy food such as seafood and too much pepper should be avoided, as well as alcohol consumption, to avoid stimulating the skin and causing itchiness and other adverse reactions. In addition, ACE has some contraindications, including coagulation dysfunction combined with infection, bleeding; local skin infection; combined with severe heart, liver and kidney dysfunction or critical illness cannot use this method.

This study is not without its limitations. First, the quality of the studies included in this research was predominantly subpar. The results of the Jadad scale indicated that only 2 of the 23 articles could be classified as high-quality studies. Furthermore, 10 articles failed to clearly delineate their randomisation methods, and only 2 employed concealed randomisation. The majority of the studies did not utilise a double-blind design; this means that the researchers were not blinded to observers, which may have resulted in both selection and observation biases. Second, the final articles incorporated in this study were exclusively domestic, which may have introduced publication bias and potentially compromised the external validity of the results. Third, differences in the baseline characteristics of patients, criteria for efficacy evaluation and variations in ACE techniques (e.g., the selection of points and depth of embedding) contributed to low homogeneity among the studies. Furthermore, the inherent specificity of ACE posed challenges in terms of fully establishing double-blind conditions, which may have compromised the reliability of the results. Consequently, the outcomes of this study have limited reference value. Additional high-quality RCTs with large sample sizes are necessary to validate the clinical efficacy and long-term safety of ACE for FC.

## 5 Conclusion

This study, which is grounded in a meta-analysis, demonstrates that ACE exhibits significant clinical efficacy in the treatment of FC. The efficacy of this method surpasses that of oral Western medicine, standard acupuncture and placebo treatments. Furthermore, it is associated with mild adverse reactions and a high safety profile. The potential mechanisms of action may include the regulation of meridians, modulation of the neuroendocrine axis, and the enhancement of gastrointestinal motility. Future high-quality RCTs are required for further validation to facilitate the clinical dissemination and application of this therapy.

## Data Availability

The original contributions presented in the study are included in the article/Supplementary material, further inquiries can be directed to the corresponding author.
