# Supplementary material for: A meta analysis of the acupoint catgut embedding in the treatment of functional constipation
Source: Front Med (Lausanne). 2025 Aug 20;12:1592220. doi: 10.3389/fmed.2025.1592220 (PMC12404950; doi:10.3389/fmed.2025.1592220)
Supplement: Supplementary file 1 [file Table_1.docx]

CNKI 639 articles


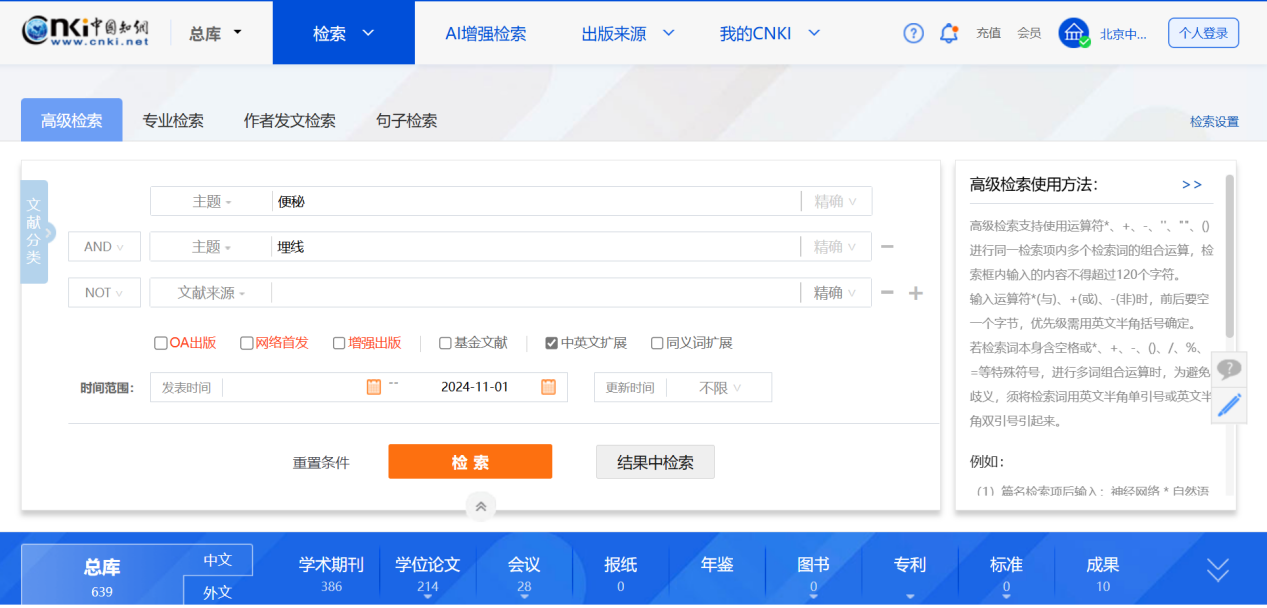


Catgut embedding

Constipation

WanFang Data 326 articles


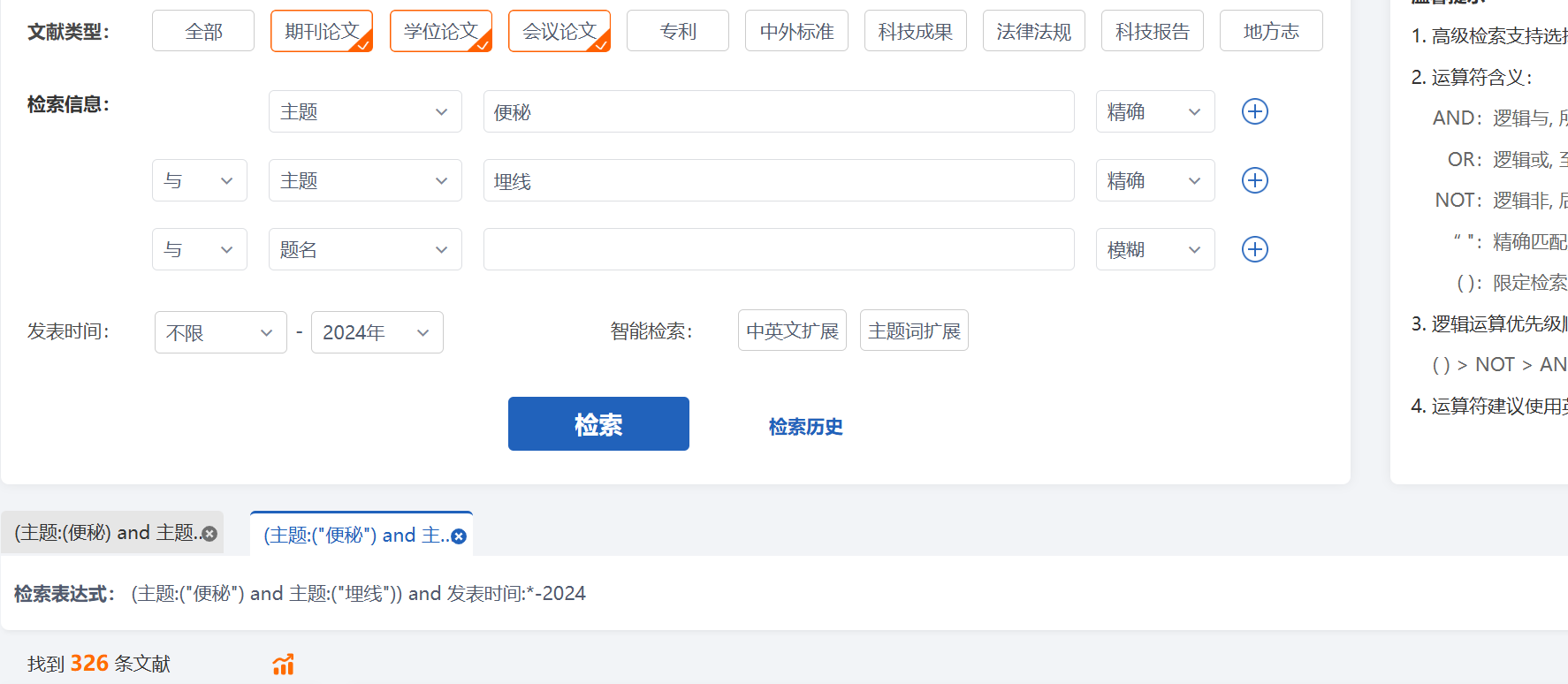


Catgut embedding

Constipation

Viper net（VIP） 85 articles


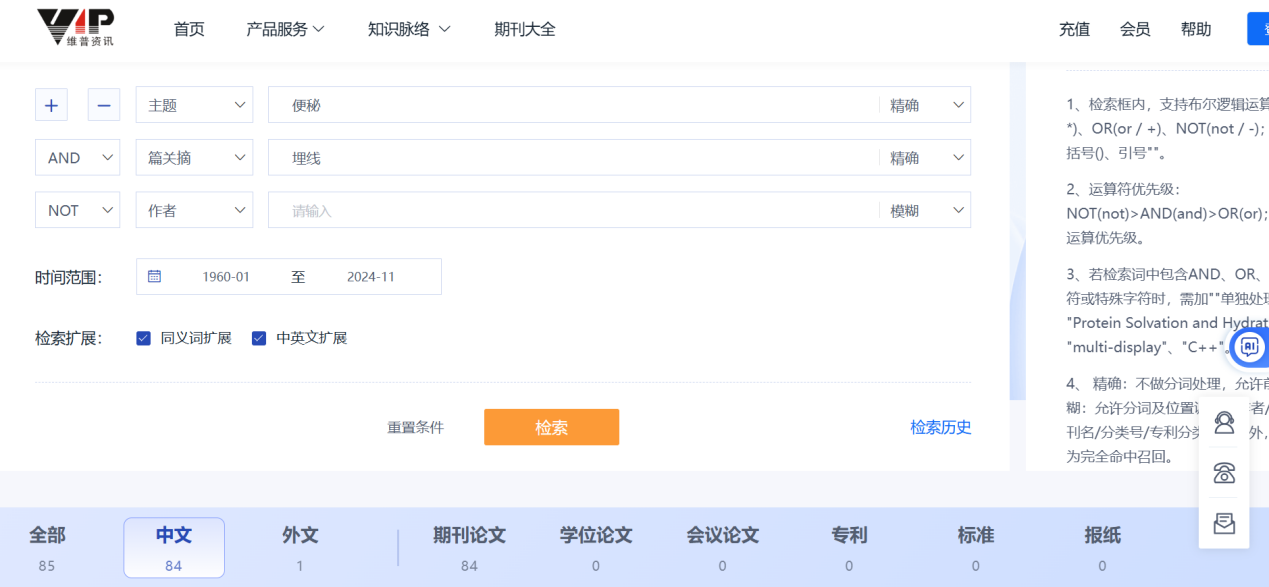


Catgut embedding

Constipation

Web of Science 39 articles
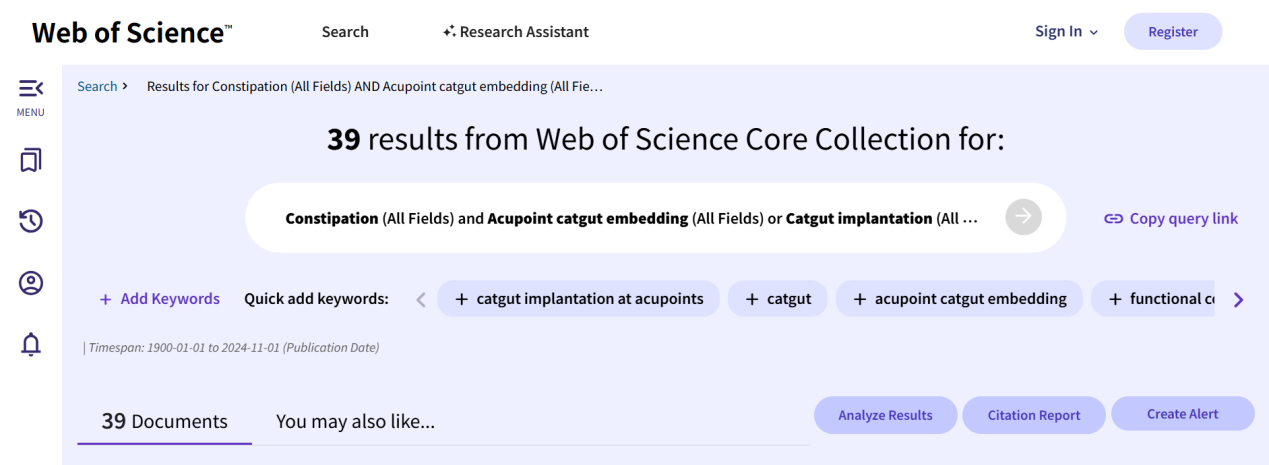


PubMed 78 articles


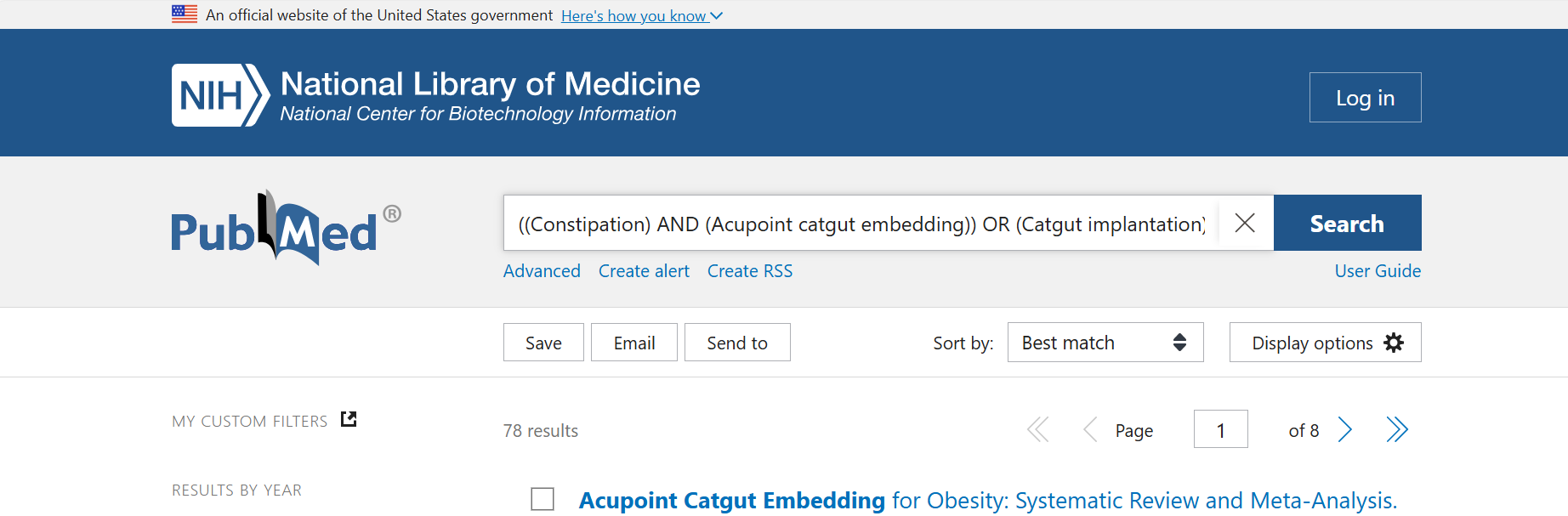


The Cochrane Library 2 articles


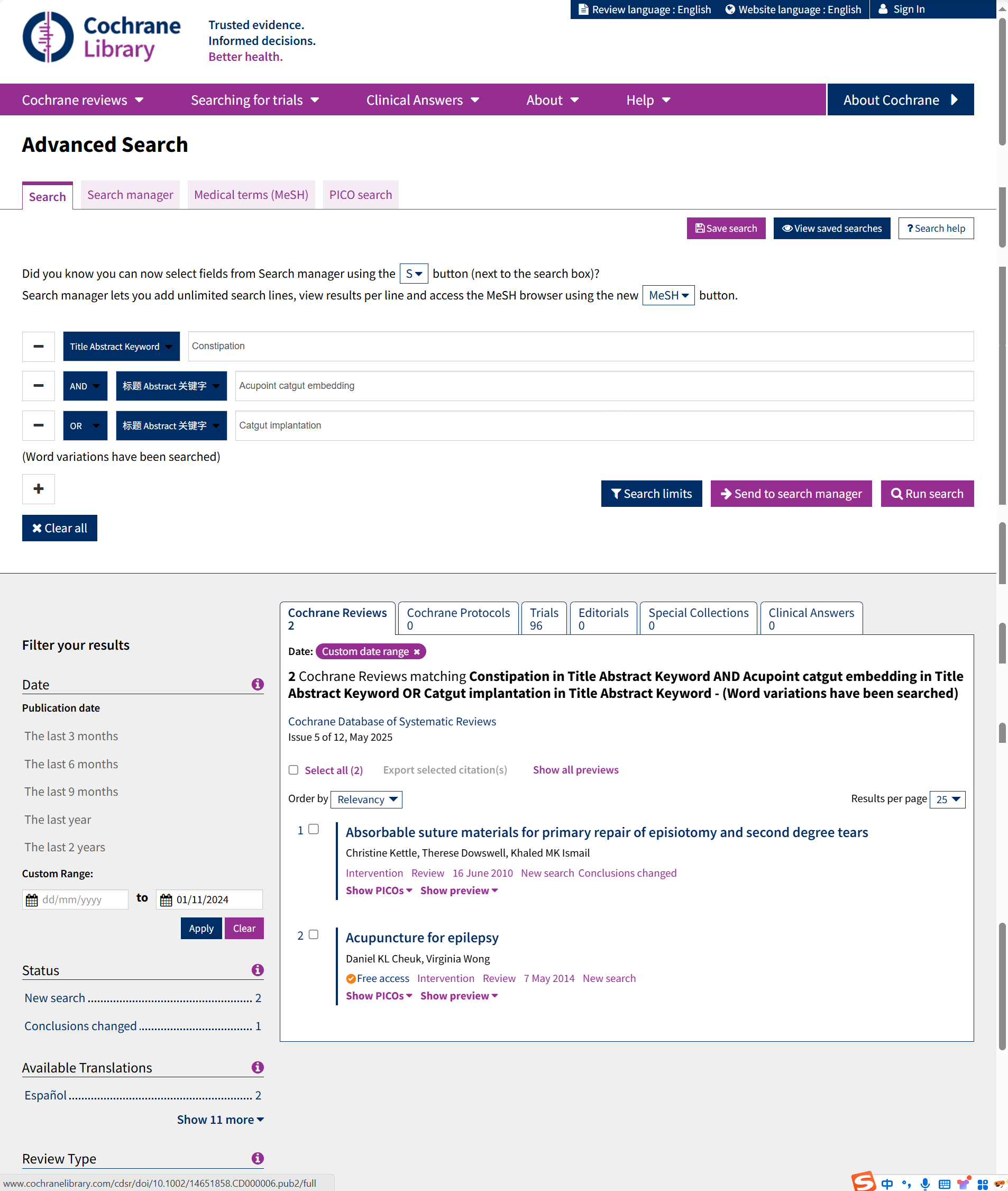


1169 total retrieved


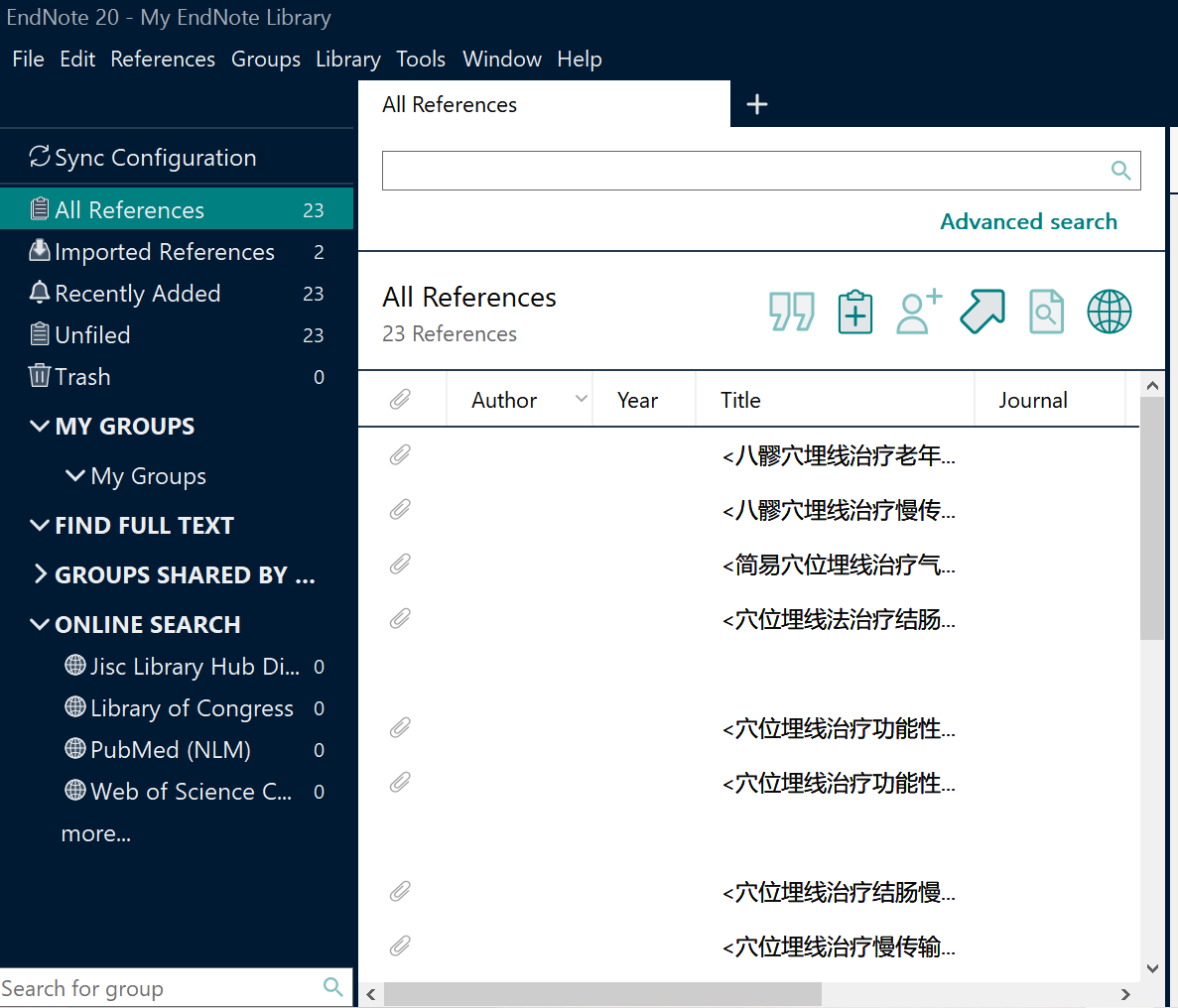
23 articles were included
