# Supplementary figures and images for: A meta analysis of the acupoint catgut embedding in the treatment of functional constipation
Source: Front Med (Lausanne). 2025 Aug 20;12:1592220. doi: 10.3389/fmed.2025.1592220 (PMC12404950; doi:10.3389/fmed.2025.1592220)

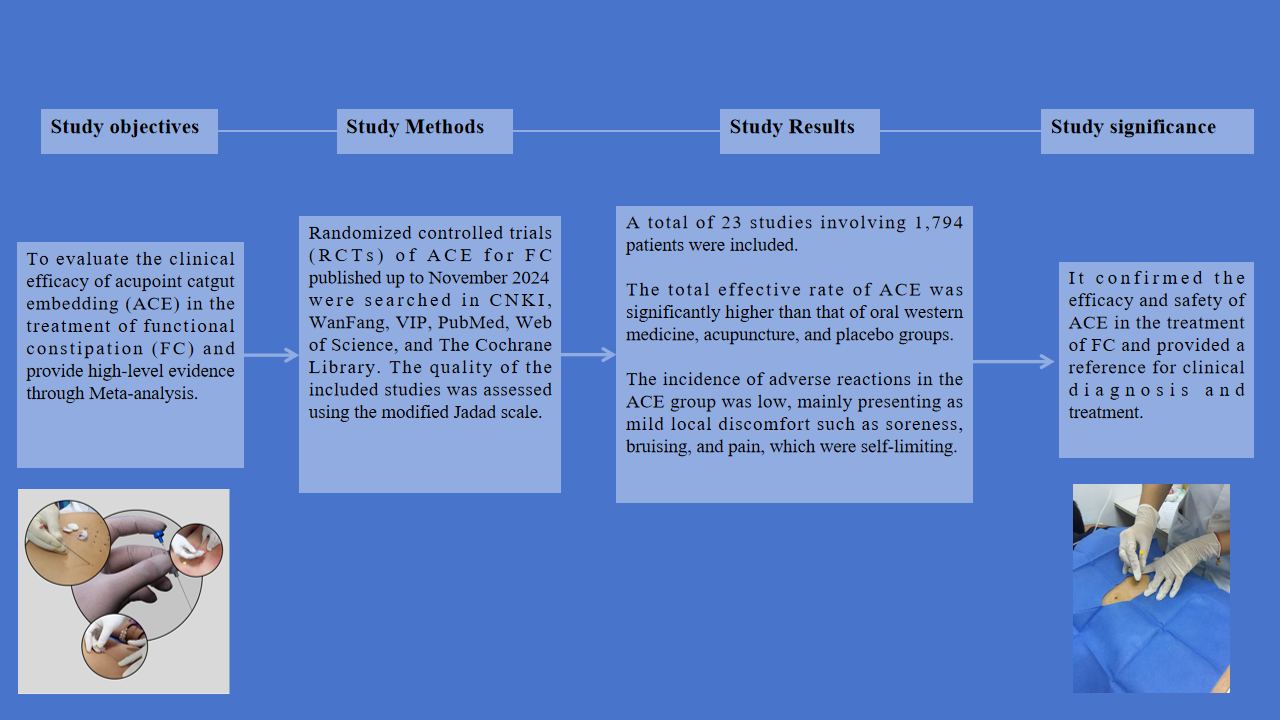

Supplement: Supplementary file 2 [file Image_1.jpeg]
